# Characterization and Potential Applications of Dog Natural Killer Cells in Cancer Immunotherapy

**DOI:** 10.3390/jcm8111802

**Published:** 2019-10-27

**Authors:** Alicia A. Gingrich, Jaime F. Modiano, Robert J. Canter

**Affiliations:** 1 Department of Surgery, University of California Davis, 2221 Stockton Blvd, Sacramento, CA 95817, USA; agingrich@ucdavis.edu; 2 Animal Cancer Care and Research Program, College of Veterinary Medicine and Masonic Cancer Center, University of Minnesota, 1365 Gortner Ave, St. Paul, MN 55108, USA; modiano@umn.edu

**Keywords:** canine, natural killer cell, immunotherapy, translational research, cancer

## Abstract

Natural killer (NK) cells of the innate immune system are a key focus of research within the field of immuno-oncology based on their ability to recognize and eliminate malignant cells without prior sensitization or priming. However, barriers have arisen in the effective translation of NK cells to the clinic, in part because of critical species differences between mice and humans. Companion animals, especially dogs, are valuable species for overcoming many of these barriers, as dogs develop spontaneous tumors in the setting of an intact immune system, and the genetic and epigenetic factors that underlie oncogenesis appear to be similar between dogs and humans. Here, we summarize the current state of knowledge for dog NK cells, including cell surface marker phenotype, key NK genes and genetic regulation, similarities and differences of dog NK cells to other mammals, especially human and mouse, expression of canonical inhibitory and activating receptors, ex vivo expansion techniques, and current and future clinical applications. While dog NK cells are not as well described as those in humans and mice, the knowledge of the field is increasing and clinical applications in dogs can potentially advance the field of human NK biology and therapy. Better characterization is needed to truly understand the similarities and differences of dog NK cells with mouse and human. This will allow for the canine model to speed clinical translation of NK immunotherapy studies and overcome key barriers in the optimization of NK cancer immunotherapy, including trafficking, longevity, and maximal in vivo support.

## 1. Introduction

The innate immune system is comprised of a spectrum of innate lymphoid cells (ILC) that are capable of mounting an immune response to pathogens and “stressed” cells in the absence of major histocompatibility complex (MHC)-restricted receptor ligand interactions [1,2]. Of the ILCs, natural killer (NK) cells are non-T, non-B lymphocytes that are known for their cytotoxicity and cytokine-producing effector functions [3,4]. Able to control microbial infections, viruses, and tumors, and being widely distributed anatomically throughout the body, NK cells are considered as the sentinels of the innate immune system. Importantly, they are able to kill targets, such as tumor cells and virally-infected cells, without prior antigen sensitization, a critical feature that has made this cell population appealing in immuno-oncology research [1,2,4,5,6,7,8,9,10,11]. 

The regulation of NK cells relies on a complex interplay of activating (e.g., NKG2D/E, natural cytotoxicity receptors, CD16 in humans) and inhibitory (NKG2A, killer immunoglobulin-like receptors in humans, and Ly49 receptors in mice) signals [1,3,8,12,13,14]. Central to the concept of NK licensing (also referred to as arming and disarming), the killer-immunoglobulin-like receptors (KIRs) in humans bind major histocompatibility complex (MHC) I, while mice use a corresponding family of receptors known as Ly49 for MHC-I binding [8,15]. When KIRs or Ly49 receptors are expressed and there is binding with MHC-I on target cells, NK cells do not initiate a cytotoxic response (although this inhibitory mechanism can be overcome when activating signals are sufficiently strong to trigger engagement of cytotoxic pathways). For this reason, when MHC-I is downregulated, which often occurs in tumors and virally-infected cells as a strategy for evading antigen specific T cell responses, NK cells lose this inhibitory signal from inhibitory KIRs and Ly49s and the threshold for NK activation/ cytotoxicity is lower [6,10,11,16]. This phenomenon has been termed the “missing-self” hypothesis and it is the cornerstone of the foundation for harnessing NK cells for cancer therapy, as it is the absence of the inhibitory MHC-I signal on licensed NK cells that underlies their cytotoxic effects to kill cancer cells [1,2,3,8,12,14]. However, when NK cells lack KIR or Ly49 expression, important immuno-regulatory functions are marshalled to prevent promiscuous/non-specific activation of NK cells against potential target cells [8,16]. This key concept underlies NK licensing, and an ongoing debate exists regarding whether NK cells gain KIR/Ly49 expression as they mature to become “armed” or they lose KIR/Ly49 expression to become “disarmed” [12,14]. 

Harnessing NK cells for cancer therapy has been a topic of research for decades. In 1985, Rosenberg et al. performed the first systemic administration of autologous lymphokine-activated killer (LAK) cells and recombinant IL-2 to patients with metastatic renal cell cancer and melanoma [17]. Of the 25 patients that were treated with this combination, a partial response was observed in 10 patients and a complete in one patient with subcutaneous melanoma metastases. However, severe toxicity was also reported and attributed to the IL-2 administration, as the authors had previously transfused LAK cells with few adverse effects. The most common, life-threatening side effect was vascular leak syndrome, which was associated with pulmonary interstitial edema, fever, chills, and malaise. A substantial fraction of patients in receiving this treatment required intensive care unit (ICU) admission to manage organ failure, and several died [17]. Since that time, research has focused on defining NK cell populations (which are now known to be distinct from LAK cells), manipulating NK cells to improve anti-tumor effects, and minimizing the side effects of the amplified cytotoxic lymphocyte responses [2,4,10,11,18,19,20,21,22,23]. 

Pre-clinical studies of human and mouse NK cells have focused on several approaches [22,23]. The infusion of NK cells following ex vivo activation and expansion are among these, as endogenous circulating NK cells are relatively under-represented in absolute numbers (especially in solid tumors) and they do not proliferate in the absence of cytokine signaling [24,25,26]. However, the limited persistence of ex vivo activated NK cells once they are adoptively transferred to the in vivo environment is a key barrier in the translation of NK immunotherapy. In vivo cytokine support after adoptive transfer of NK cells, much like that attempted by Rosenberg, has been evaluated for other members of the interleukin family that are known to stimulate NK cells, including IL-12, IL-15, and IL-21 [24,25,26,27,28,29]. Cytokine infusion via bolus intravenous dose of IL-15 has been attempted in humans with some clinical success, however at a low maximum-tolerated dose due to dose-limiting toxicities [30]. Gene modification of the NK cells to express chimeric antigen receptors, which is analogous to what is done in T cell therapy, is also being explored in preclinical and clinical studies [31]. However, these approaches are challenging because primary NK cells are hard to stably transduce and concerns exist regarding the neoplastic potential of genetically modified transformed NK cells (such as the prototypical NK-92 line), which have been shown to be amenable to genetic manipulation [32]. As NK cells have a multitude of inhibitory receptors, research into methods to block such receptors is underway utilizing the current checkpoint blockade with PD-1/PD-L1 inhibitors as well as other novel and potentially NK-specific targets [33]. Many of these methods are currently in preclinical development or early stage clinical trials (NCT03937895, NCT03958097, NCT03815084). However, the extent to which these approaches are NK-specific (given the overlap of NK cells with cytotoxic T cells) is an important caveat.

The preclinical mouse model (*Mus musculus*) has been invaluable in advancing our understanding of cancer immunology and cancer immunotherapy. However, the intrinsic characteristics of murine models create challenges for the application and translation of these results to the clinical setting [34]. Carcinogen-induced murine models lack the accumulation of genomic events that lead to neoplastic transformation that are seen in human cancers. Although driven by known driver-oncogene mutations, genetically engineered murine models do not recapitulate the genomic diversity of spontaneous tumors or the complex epigenetic environment of the tumors or the microenvironment in which they exist. Patient-derived xenograft (PDX) murine models, which do recapitulate tumor heterogeneity and critical tumor microenvironmental factors, nevertheless lack an intact immune system that is critical in assessing immune surveillance and immune evasion phenomena. Even among syngeneic, immune component murine models, there are still important gaps in the modeling of immune elimination, equilibrium, and escape, elements that are considered to be fundamental in cancer’s adaption to immune surveillance [34]. In human cancer, NK cells are considered to be most relevant in the early phases of immune editing, in which the NK cells drive the elimination of immunogenic tumor cells and shape the composition of remaining tumor cells that are present for the periods of elimination and escape [4,12,14]. This step is lacking in standard mouse models of cancer due to the use of transplantable mouse tumor lines and/or genetically engineered models (GEM), neither of which are truly naturally occurring as happens with humans who spontaneously develop cancer over many years in the setting of an intact immune system. Therefore, a model that parallels the heterogeneity and complexity of human cancers, as well as the critical interactions with the immune system, would facilitate the translation of novel immunotherapies, especially NK immunotherapy, given the key species differences between mice and humans as noted above. 

Several features of dogs (*Canis lupus familiaris*) make this species an attractive candidate for immunotherapy research in general and NK immunotherapy in particular. Unlike inbred mice, dogs are large and outbred, providing a test population with greater genetic diversity. Dog cancers spontaneously develop in the setting of an intact immune system. While genetic analysis of some dog cancers has demonstrated homology to human cancers, including pathogenic translocations of Bcr-Abl, and conserved mutations of BRAF and c-kit, [11,35,36,37,38,39,40], perhaps the most important aspect of the canine model is the increasing incidence that is seen with age. Dogs are the only species besides humans where individuals exceed the evolutionarily-determined constraint on longevity, inevitably creating cancer-prone phenotypes [41]. Additionally, dogs are companion animals and, as a result, they are subject to many of the same environmental conditions and epigenetic influences as their human counterparts [37]. For example, previous work has demonstrated concordance between the microbiomes of humans and their dog companions [42]. Intriguingly, various immune-mediated skin diseases, such as psoriasis and eczema, also have a pet-owner association [39,43]. When studying the complex and context-specific interplay of tumors, the immune system, and the environment, few species are so well suited to simulate the human condition as companion dogs [34,35,36,37,39,40,42,44]. 

At the total population level, companion dogs are also much like their human counterparts: diverse and outbred. However, paradoxically, from a population genetics perspective, when compared to the general dog population as a whole, specific dog breeds are conversely highly inbred and prone to an increased incidence of specific anomalies, including congenital birth defects and breed-specific cancers (among other diseases) [39,40]. It is possible that differences in immune surveillance could contribute to this inter-breed variation in incidence and type of cancer. Evidence for the diversity in NK cell repertoires and their implication in human viral illness has been studied, and it is known that NK cell deficiencies or genetic mutations place human patients at a higher risk for malignancy [45,46,47,48]. While not yet pursued in canine research, the similar population and subpopulation structure of breed variation might lend itself to the study of the impact of NK cell repertoires on cancer development, response to treatment, and immune responses. Additionally, as with clinical trials, with greater coordination and infrastructure, dog studies lend themselves to population studies that can be highly informative regarding the impact of immune surveillance on oncogenesis, given the ability to select breeding partners, increased progeny, and shorter generation time [49]. 

In both humans and mice, NK cells originate from the oligopotent common lymphoid progenitor (CLP), which also gives rise to B cells, T cells, and non-NK cells [1,2,22,50]. Murine NK cells undergo development in the bone marrow, as do human NK cells, but, importantly, human NK cells subsequently mature in a variety of secondary lymphoid tissues, including lymph nodes, spleen and tonsils [51]. As outlined in a recent review by Abel et al, murine NK cells move from refined NK-cell precursors (rNKPs) to immature NK (iNK) cells with the acquisition of NKG2D cell surface receptor [15,50]. NK cells then move through six developmental stages (Stages A–F) before reaching maturity that is characterized by the expression of killer cell lectin like receptor G1 (KLRG1). Human NK cells also undergo six stages of development. Differentiation completes in Stage 6 with the expression of KIR and CD57, among other cell surface markers [50]. 

In humans, the cytotoxic properties of NK cells have inspired comparisons to CD8^+^ T cells [52]. NK cells have key differences, although there are clear similarities in phenotype and function. One important distinction is lifespan, as NK cells are classically considered to have an “intermediate” life span on the scale of leukocyte longevity (although this remains an unresolved question given the difficulty in performing limiting dilution and clonal expansion of single NK cells) [50,51]. Recent work has elucidated further comparisons between cytotoxic T cells and NK cells with the discovery of induced NK cell memory or adaptive NK cells. In these key studies, NK cells have demonstrated secondary responses, including recall towards specific antigen, such as (murine) cytomegalovirus (CMV/MCMV), human immunodeficiency virus (HIV), influenza viruses, and haptens [47,53,54]. Following such an exposure, Stage 6 NK cells appear to acquire an extended life span with the ability to self-renew in what was previously considered to be the stage of terminal differentiation [47,53,55,56]. 

The surface markers and phenotypes at various stages of differentiation for dog NK cells have not been defined to the point of organizing development into stages, although recent research has begun to assess maturity based on the density of CD5 receptor expression [57]. Given the persistence of T cell markers, such as CD5 and CD3 on putative NK cell populations, it is reasonable to postulate that dog NK cells are closely related to their T cell counterparts, as is seen in humans. Like effector T cells, dog NK cells are functionally defined by their cytotoxic properties and the preservation of this phenotype is used to validate candidate surface markers that are thought to represent canine NK cells populations [57,58,59,60,61]. No classical adaptive characteristics, such as the secondary responses and memory following viral exposure, have been explored in dogs to date.

Of significant interest for NK biologists, a canine variant of CMV that afflicts dogs has not been identified, although dogs are exposed to canine distemper virus (CDV), which does activate NK cells [62]. However, it is exposure to CMV/MCMV that is thought to have driven the genetic expansion of the KIR and Ly49 genes in humans and mice, respectively, and the absence of this evolutionary driver might have implications for canine NK cells [63]. It has been shown in mice, for example, Ly49H^+^ NK cells expanding following MCMV infection and inducing a long-term, or “memory”, NK cell response [55]. A similar effect was demonstrated in humans following CMV reactivation after allogeneic transplant, which resulted in increased expression of the NKG2C receptor, a member of the KIR family [64]. The viral exposure-NKG2C association was tested, and it appears only that CMV has durable effects of the human NK cells repertoire, as positive serology for EBV and herpesvirus was not associated with increased expression [65]. CD57, a marker of human NK cell maturity, is highly expressed in NKG2C^+^ cells post-CMV exposure, whereas inhibitory NKG2A has decreased expression [66]. Notably, few studies have examined the phenotype and function of dog NK cells in the context of viral infection, and none have addressed the development of memory or adaptive response [62,67,68]. As CMV-specific infection, seems to be critical in the acquisition of the NK cell memory phenotype, especially in humans (based on the evidence to this point); it is interesting to speculate that canine NK cells may have decreased or insufficient capacity to acquire a “memory” or “adaptive” phenotype in the absence of a CMV-equivalent. Future studies on this topic will shed important insight into NK biology.

## 2. The Genetic Basis of Canine NK Cells

While the populations of innate lymphoid cells have been extensively studied in humans and mice, detailed studies of this nature are lacking in dogs, and, currently, dog NK cells are relatively poorly defined [69,70,71,72]. Human and mouse NK cells have inhibitory receptors that inhibit NK responses to cells bearing the MHC-I marker [73]. These inhibitory receptors are the crux of the so-called “missing-self” hypothesis, in which NK cells are stimulated to respond to targets when activating receptors are engaged in the absence of MHC-I/inhibitory receptor binding. Human NK cells have both inhibitory and activating MHC-I-specific KIR receptors [8]. The inhibitory KIR receptors act through the intracytoplasmic immunoreceptor tyrosine-based inhibition motifs (ITIMs) to recruit tyrosine phosphatases, such as SHP-1 and SHP-2 [14,73]. Murine NK cells have lectin-like Ly49 dimers as their surface MHC-I binding receptor. The Ly49 dimers in mice also possess intracytoplasmic ITIMs, which facilitates inhibitory function (although there are Ly49 isoforms that are also activating in mice such as Ly49D and Ly49H) [12,73]. A third MHC-I inhibitory receptor, the CD94-NKG2A heterodimer, is present in both species [1,8,12,14]. Key steps in regulating NK activation occur based on the integration/summation of the activating and inhibitory signal inputs from all of these receptors. This mechanism of NK activation or inhibition appears to be different from T cell signaling, which is antigen specific, MHC-restricted, and is predicated on sequential signals (T-cell receptor (TCR) cross-linking, co-stimulation, cytokine exposure), which if not all present lead to anergy and/or senescence [14]. NK cells fluctuate on the summation of inhibitory and activating receptor signals and this paradigm for NK activation is conserved in mice and humans. This is a critical difference between NK cells and cytotoxic T cells, although there is evidence that bystander CD8^+^ T cells can be activated (and fluctuate in activity) based high dose cytokine exposure in the absence of antigen specific signals [74]. Although studies have yet to address the precise mechanisms of NK activation and inhibition in dogs, it has been presumed in the literature that these mechanisms are evolutionarily conserved [60,75]. 

As discussed above, KIR and Ly49 gene sequences encode polymorphic NK cell receptors that have either activating or inhibitory roles. Genetic selection/evolution likely plays a role in the structural diversification of the receptors given the multitude of ligands. However, the similarity in function for KIR-bearing cells in humans and Ly49-bearing cells in mice despite structural differences in the receptors has led some to postulate a role for convergent evolution that is driven by MHC [63]. Genomic analysis suggests that the expansion of the KIR receptors in humans appears to have over-ridden the evolution of human Ly49 genes [63]. The existence of the Ly49 gene has been reduced to a single pseudogene in humans, and significant differences exist between the murine and human NKC-Ly49 gene regions. The NK gene complex (NKC), which governs C-type lectin-like CD94-NKG2A heterodimer MHC-I receptors, has been extensively studied in humans and mice [63,76]. Importantly, the NK gene complex has also been detected in species ranging from fish to humans. The NKC appears to be conserved in terms of position, structure, orientation, order, and homology, but it is heterogeneous for gene content [63,77]. In particular, a key conserved feature among species is a high linkage for NK lectin-like receptor genes and C-type lectin receptor genes. However, the dynamics of the NKC lend itself to gene duplications, expansions, and conversions, as seen with the Ly49 expansion in mice [63,76]. 

Where, then, do the canine NK MHC-I receptors fall on this scale? Despite the completion of sequencing the dog genome, few studies have directly examined this question. Hao et al. examined the NKC in six mammalian genomes, including dog. The NKC in dogs resides on a single region on chromosome 27, with adjacent regions for group V and group II proteins [77]. Of the C-type lectin genes in the NKC, the dog genome codes for group V proteins (that function as NK cell receptors for MHC-I molecules) and group II proteins (not expressed on NK cells). In terms of gene length, the dog, human, and mouse NKC are 2.4Mb, 2.8Mb, and 8.7Mb, respectively. The dog has 22 NKC genes, as compared to humans (29 genes) and mouse (57 genes). Orthologs of the killer cell lectin like receptor (KLR) genes, particularly KLRD1 (CD94), appear to be highly conserved between human, dog, and mouse based on comparative genomic analysis [63,72]. However, it is important to note than the inhibitory companion protein, NKG2A, which forms a heterodimer with CD94 in humans and mice to recognize MHC-I, has not been studied in canines and it is not annotated on the latest canine genome assembly CanFam3.1 [78]. Phylogenetic analysis supports the rapid expansion of the mouse NKC that is caused by a doubling of the KLR- and C-type lectin (CLEC)-type genes. KLRA, CLEC2D and CLEC4/A/B/C specifically underwent the greatest expansion [77]. Of note, KLR genes are expressed on NK cells and they may have expanded in response to repeated viral infection, while CLEC have no proven role in NK cell function [76]. 

Analysis of the dog genome for evidence of Ly49 and KIR genes has also been partially studied. In mice, Ly49 is considered to be a multi-gene family. Gagnier et al. determined that dogs have one copy of the Ly49 gene while using the Southern blot technique, as is the case with cat (*Felis catus*) and pig (*Sus scrofa*) [79]. The Ly49 gene in dogs is located in the NKC on chromosome 27, and subsequent analysis determined that this protein contains an ITIM domain [79]. However, it was also noted by these authors that the dog Ly49 gene has a cysteine-to-tyrosine conversion at position 168. Within the Ly49 gene, there are normally six highly-conserved cysteine residues in the C-type lectin-like domain that is involved in disulfide bonds. Without this cysteine, the capability of the dog Ly49 gene to function is unclear, prompting Gagnier et al. to hypothesize that the dog Ly49 gene was non-functional. However, further evaluation to verify these findings has not been published at the time of this manuscript [79]. Interestingly, a cysteine-to-phenylalanine Ly49 gene mutation is also present in the pig at position 168. However, it is important to note than this study (published in 2003) utilized Southern blot analysis, based on the known exon-intron structure of mouse genes, to identify these genetic polymorphisms. Therefore, the current techniques of next-generation sequencing may shed new light on these findings and allow for a re-examination of whether these genes encode functional or non-functional NK proteins. It is also conceivable that other Ly49 genes may exist in dogs that differ too greatly from the probes that were used at the time, and thus prevented detection [79]. Similarly, Hammond et al. studied the KIR gene family in mammals [80]. The KIR gene typically lies in the leukocyte receptor cluster (LRC, on chromosome 1 in dogs) between the leukocyte immunoglobulin-like receptors (LILR) and Fc fragment of IgA receptor (FCAR) genes in a highly variable region. Based on genome build 2.1 assembly for dogs (current assembly is genome build 3.1), the KIR gene appeared to be absent and the FCAR gene prematurely truncated at the 5’ end [78,80]. Based on this evidence, the authors concluded that dogs lack a functional KIR gene [80]. However, gaps in genomes, particularly in highly variable or repetitive regions, are a known error for genome assemblies, and this finding has not been validated with long-read technology. This raises the possibility that further analysis might, in fact, reveal a functional KIR gene in dogs that would have key implications for NK biology in dogs. 

## 3. Phenotypic Identification of Canine NK Cells with Surface Markers

Defining phenotypic surface markers to identify canine NK cells has been a longstanding topic of investigation. In humans, NK cells are distinguished at their most basic level by the absence of CD3 and the presence of CD56 [81]. Furthermore, human NK cells have been classified by levels of CD56 expression with the CD56^bright^ sub-population generally co-expressing muted levels of CD16 and exhibiting greater cytokine production, with the CD56^dim^ expressing high levels of CD16 and possessing potent cytotoxic function [81]. NK maturation and tissue specific NK phenotypes (as well as distinctions from other innate lymphoid cells) are key areas of investigation for both human and mouse NK biology [1,81,82]. Murine NK cell surface markers vary, depending on mouse lineage and strain. C57BL/6 and Swiss Jim Lambert (SJL) mice express NK1.1, CD49b, and NKp46 [81]. These cells must also be CD3 negative to classify them as NK cells, as CD3 is a marker of T cells and expression is not exclusive to NK cells, but is context dependent. The BALB/c mouse strain possesses too many allelic variants for NK1.1 to render this a reliable and detectable marker. Therefore, BALB/c NK cells are defined by the presence of CD49b and NKp46 [81]. The phenotypic characterization of dog NK cells, while not understood to the depth of human and mouse NK cells, has been the topic of recent studies and it is improving. 

Morphologically, dog NK cells are medium-sized to large lymphocytes with electron-dense intracytoplasmic granules that contain granzyme B and perforin [83]. They are CD4-/CD20-, as these are phenotypic markers for T and B cells, respectively [83]. However, as with human and, to a lesser extent, mouse NK cells, CD8 expression is often observed on putative canine NK cells, with the expression in some conditions reportedly encompassing as much as 30% of the population [61,84]. In dogs, NK cell candidate populations are also cytotoxic against known triggers without MHC restriction, such as canine thyroid adenocarcinoma cells (CTAC), a cell line that is commonly used to test NK effector function [84]. For example, Huang et al. described such an NK population isolated from canine peripheral blood based on low expression of the surface marker CD5, the so-called CD5^dim^ subset [85]. CD5 is a member of the scavenger receptor cysteine-rich superfamily and is typically classified as a T cell marker [86,87]. In fact, CD5^bright^ cells in the dog have been classified as T cells. The active domain of the CD5 protein is highly conserved and is thought to be involved in basic homeostatic functions, such as calcium signaling and innate immune defense [85,86]. The CD5 gene lies on chromosome 18 in dog and it is conserved in human and mouse [78]. Akin to the CD56 marker in humans, Huang et al. noted different characteristics of lymphocytes based on density of CD5. CD5^dim^ cells comprised about 15% of isolated peripheral blood mononuclear cells (PBMCs) and were morphologically larger than CD5^bright^ cells [85]. Following exposure to IL-2, CD5^dim^ cells contained more cytoplasmic granules and demonstrated antigen-independent cytotoxicity. PCR revealed elevated levels of mRNA for several NK receptors and activation markers including NKp30, NKp44, CD16, and CD94 [85]. This study was the first to suggest that CD5^dim^ is a candidate phenotypic marker for dog NK cells, especially in the setting of expansion/ activation using IL-2 enrichment [85]. 

Shin et al. expanded cytotoxic large granular lymphocytes (CLGLs) over 14 days in media containing a combination of human IL-2, IL-15, and irradiated K562 feeder cells [58]. The authors noted that the proportion of the dog morphologic CLGLs increased as compared to PBMCs over the 14 days of exposure to human cytokines, providing evidence of cross-reactivity of dog lymphocytes with human cytokines [58]. In addition, phenotypic analysis of surface markers on CLGLs was performed while using multi-parameter flow cytometric analysis following expansion. The majority of the CLGLs were of the CD5^dim^CD3^+^CD8^+^TCRαβ^−^TCRγδ^−^CD4^−^CD21^−^CD11c^+^/^−^CD11d^+^/^−^CD44^+^ phenotype. CD3 and CD8 are traditional T lineage markers, but cells were noted to be lacking the TCRαβ and TCRγδ T cell receptors that were seen on all T cells [58]. The CD3^+^/iTCRα^+^ phenotype had been previously described by Yasuda et al. and was felt to represent an NKT cell population, although this population was isolated from T lymphocytes, suggesting possible plasticity in the lineage [87] CLGLs isolated in the Shin study only had trace amounts of iTCRα and, thus, despite occurring in the setting of a CD3^+^ phenotype, were thought to be NK cells and not NKT cells. 

Follow up studies evaluated the phenotypic differences between the CD5^dim^CD3^+^CD21^−^ putative NK cell population and non-B non-T large granular lymphocytes, CD5^−^CD3^−^ CD21^−^TCRαβ^−^TCRγδ^−^GranzymeB^+^ [57] CD5^dim^CD3^+^CD21^−^ cells were isolated by flow cytometry and expanded in culture containing IL-2, IL-15, IL-21, and K562 feeder cells for 21 days. As time elapsed, the frequency of CD5^dim^CD3^+^CD21^−^ cells decreased and the CD5^−^CD3^−^CD21^−^ cells increased, with a small proportion of CD5^dim^CD3^−^CD21^−^ cells remaining [57]. Importantly, after ex vivo co-culture, the expanded cells did not express TCRαβ or TCRγδ, which was considered to be evidence for expansion of NK cells. CD3^+^CD5^dim^CD21^−^ cells exhibited significantly higher IFN-γ cytokine production when compared to CD3^−^CD5^−^CD21^−^ cells. Cytotoxicity was not significantly different between the two populations, although CD5^dim^CD3^+^CD21^−^ had significantly higher levels of NK surface markers, including IFNγ [57]. The relatively rapid phenotype switching in culture was attributed to activation, and the authors proposed that each population represents putative NK cells at different degrees of maturation (based on levels of the T-box transcription factors T-bet and Eomes), with CD5^−^CD3^−^CD21^−^ cells nearing exhaustion after 21 days in culture [57]. 

Another important dog NK phenotypic marker is NCR1/NKp46, which is an NK-specific activating molecule. NKp46 is widely considered to be a “pan-species” NK marker for mammals and it has been identified on human, primate, rat, mouse, ovine, and bovine NK cells [8,88]. However, for unclear reasons, expression in pig NK cells appears to be diminished [89]. With respect to canines, although the KIR genes in the canine leukocyte receptor cluster (LRC) appear to be absent, the canine genome otherwise has homologous expression of natural cytotoxicity receptors with humans and the NCR1 gene is present in the LRC on chromosome 1 in dogs [77,78,80]. Studies by Grondahl-Rosado et al. examined the populations of CD3^−^GranzymeB^+^ cells, which were NCR1^+^ and NCR1^−^ [70,71]. They noted that the proportion of CD3^−^NCR1^+^ cells in PBMCs was markedly lower than expected, up to 2.5% of lymphocytes, when compared to the frequency of NK cells observed in human and mouse blood. This percentage is also low in relation to the frequency of CD5^dim^ cells in dog blood, which comprises up to 15% of lymphocytes [70,85]. Following ex vivo exposure to both human and canine IL-2 and IL-15, the percentage of CD3^−^NCR1^+^ cells substantially increases. In addition, after exposure to human IL-12, NCR1^+^ expression was nearly uniform on all GranzymeB^+^ cells [71]. The authors concluded that the NK cells are comprised of both CD3^−^GranzymeB^+^NCR1^−^ and CD3^−^GranzymeB^+^NCR1^+^ populations, wherein the latter subset represents an activated population, and that the presence of NKp46 as a cell surface marker might be inducible upon cytokine exposure [71]. Based on this evidence, the use of NCR1 as a phenotypic marker to define resting canine NK cells that were isolated from PBMCs may not identify all NK cell subtypes, at least in the resting state [71]. On the other hand, although CD5^dim^ might be more sensitive for identifying greater numbers of circulating NK cells, this marker appears to identify a more heterogeneous population, which likely includes non-NK cell constituents. Further characterization of these cell populations is needed, and high throughput sequencing and/or single cell sequencing strategies may be informative, especially given the variability in single candidate dog NK marker expression between the resting and activated states. 

Of note, Foltz et al. developed a novel antibody to canine NKp46 for use in flow cytometry, alleviating what is a common hurdle in canine NK studies, which is lack of species-specific monoclonal antibodies [69]. Their work also identified CD3^−^NKp46^+^ and CD3^−^NKp46^−^ NK subsets. Once again, the CD3^−^NKp46^+^ population comprised approximately 2–3% of PBMCs, and these cells were found to be highly cytotoxic against multiple canine cancer lines, including CTAC and immortalized dog osteosarcoma tumor cell lines [69]. The CD3^−^NKp46^−^ population was less cytotoxic, but it was similar in terms of cytokine secretion. Furthermore, in using a novel expansion method that markedly increased yields to a clinically promising level, the authors showed that the CD3^−^NKp46^−^ population could be induced to express NKp46 [69]. A CD3^+^TCR^+^NKp46^+^ population was also identified. The CD3^−^NKp46^+^ and CD3^−^NKp46^−^ populations were compared to CD3^+^ T cells and were noted to be similar for cytokine secretion, but even the CD3^−^NKp46^−^ population had higher cytotoxicity compared to CD3^+^ T cells, with CD3^−^NKp46^+^ being the most cytotoxic [69]. The significance of the CD3 markers on certain populations of candidate NK cells remains an enigma, and there are several explanations as to why this traditional T-cell marker is seen. Canine NK cells could express CD3 at certain stages of maturation, as suggested by the Lee and Yasuda studies [57,87], or it could remain constitutively expressed on a small subset of lymphocytes not of the NK cell lineage that have persisted in culture, such as LAK cells. It may also be a technical issue, a non-specific artifact of antibody binding, as can occur with the use of cross-species antibodies. Regardless, evidence for the expression of CD3 on canine NK cells is not yet conclusive or completely described. 

A third candidate marker for dog NK cells is C-type lectin-like CD94 (KLRD-1). In humans, this forms a heterodimer with NKG2A and binds MHC-I. No functional studies to confirm the analogous purpose have been performed in dogs, although, as noted above, genetic analyses demonstrate high conservation of the KLDR1 gene in the NKC, but the absence/lack of annotation of the NKG2A gene [63,77,78]. Experiments have demonstrated that anti-human CD94 antibodies cross-react with canine leukocytes, suggesting conserved surface structure [72,90]. Recently, a canine-specific anti-CD94 (anti-caCD94) was developed. In this study, anti-caCD94 bound to approximately 7.7% of isolated PBMCs, placing this yield between that of CD5^dim^ (~15%) and NKp46^+^ (~2.5%) [72]. When anti-caCD94 was used on a CD5^dim^ population, two subsets emerged: CD5^dim^CD94^+^NKp46^+^CD3^−^ and CD5^dim^CD94^+^NKp46^+^CD3^+^, both of which were CD4^−^ and CD21^−^. The latter CD3^+^ population was felt to represent canine NKT cells, although there was no interrogation for an iTCRα receptor, as in the Shin and Yasuda studies [72]. The CD94^+^ cells were noted have higher cytotoxicity than resting PBMCs; however, the binding of this antibody to CD5^dim^ NK/NKT cells did not result in the augmentation or inhibition of the cytotoxic response, as measured against CTAC cells [72]. When CD94^+^ cells were expanded in a medium containing IL-2, IL-15, and an irradiated layer of CTAC cells, CD5^dim^ expression disappeared, as is seen on expanded NKp46^+^ cells. Phenotypic analysis with PCR showed expression of CD16, CD56, and Eomes on expanded cells [72]. 

**Figure 1:**
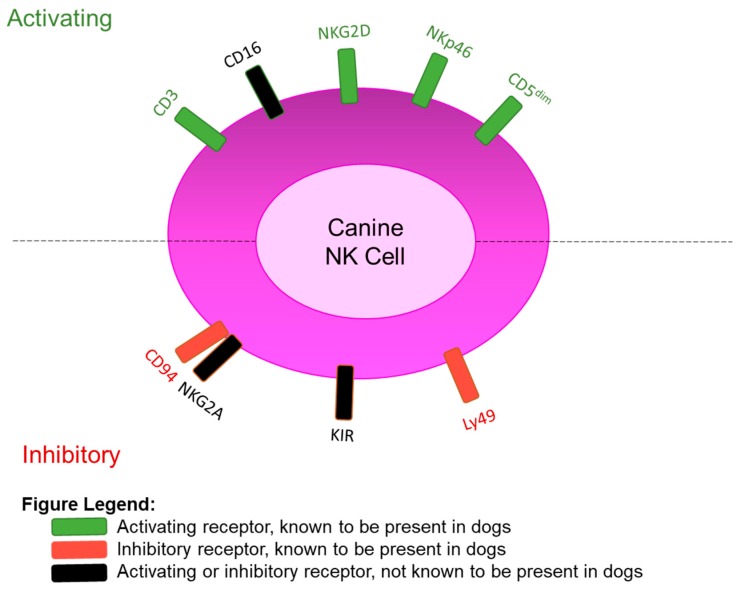
Phenotypic Surface Markers of Canine NK Cells based on Current Evidence.

CD16 is an activating receptor on human NK cells, and is widely considered one of the most potent mediators of NK cytotoxic function [8,91] Also known as FcγRIIIa and being encoded by the *FCGR3A* gene, the CD16 molecule contains a constant region of the Fc receptor. The binding of Fc portions of antibodies to the Fc receptor on NK cells triggers antibody-dependent cellular cytotoxicity, which is a critical additional mechanism that NK cells can use to kill target cells [92]. A homologous receptor has been found in mice, termed CD16-2 [93]. Studies to date have not conclusively demonstrated whether CD16 is expressed on dog NK cells, and notably the *FCGR3A* gene is not annotated on the CanFam3.1 dog genome [78]. NKG2D is another critical surface marker on NK cells (and bystander T cells), which mediates cytotoxicity. NKG2D is a prototypical NK activation marker on human and mouse NK cells, being encoded by KLRK1, which is used by NK cells to recognize and kill target cells that expressed NKG2D ligands [8,91]. These proteins are normally expressed at low levels on the surface of normal cells, but when cells are infected, transformed, and senescent (as well as rapidly proliferating cells), the expression of these induced-self proteins is upregulated. Although the KLRK1 gene has been identified on chromosome 27 in dogs with high homology to humans and mouse [77,80], the expression of the protein receptor has not been formally detected on canine NK cells at this time. Therefore, CD16 and NKG2D may be absent on dog NK cells (which would be unexpected given what is known about NK biology), or these putatively fundamental NK markers may simply not cross-react with available human monoclonal antibodies, thus precluding detection. Future studies addressing these questions will also help to advance the field of dog NK biology. Current knowledge of dog NK receptors are summarized in Figure 1 and Table 1. 

## 4. Ex vivo Manipulation and Expansion of Canine NK Cells

Based on the available data, phenotypic analysis of dog NK cells place their yield between 2.5–15% of PBMCs from resting conditions [69,71,72,85] However, the absence of a definitive NK surface marker and the relative rarity of NK cells in the circulation present obstacles to clinical translation for the use of NK cells in canine immunotherapy studies. Therefore, methods for expanding purified NK cell populations have been studied to include cytokine exposure and co-culture with feeder cell lines. As is common with canine experiments, human cytokines are often used (primarily because of access and ability to scale up for in vivo use), and investigators have successfully used recombinant human IL-2, IL-12, IL-15, and IL-21 in canine studies [9,24,25,26,27,29,58,94,95]. In addition, the irradiated K562 cell line, a chronic myelogenous leukemia tumor line derived from a human patient, is also used to expand and activate dog NK cells in culture [58,96]. The use of a virus infected cell line, such as Epstein-Barr virus-transformed lymphoblastoid cell lines used in humans, has also been attempted in canines, but with less reliable and reproducible results. 

Michael et al. described an isolation and expansion approach, in which non-T, non-B lymphocytes were isolated from PBMCs by CD5 depletion via immunomagnetic separation [95]. This method depletes T cells, which is critical for two reasons. First, T cells will expand under the same cytokines to a degree that overwhelms the NK cell population. Secondly, T-cell derived lymphokine activated killer (LAK)/NKT cells contribute to cytotoxicity behavior in the NK functional assays, as this population displays a similar phenotype, thus skewing the results [95]. This method allows for B cells and macrophages to remain, as these populations theoretically enhance the expansions of NK cells in vitro. The remaining CD5-depleted cells were then exposed to human IL-2, IL-15, IL-2/IL-15 combination or the feeder cell line EL08-1D2, which is derived from murine embryonic liver cells, instead of human cancer cells, like the K562 line [95]. Of note, the authors found that, in the IL-15 only groups, fewer than 10,000 cells survived after 14 days. The maintenance of cell numbers was seen in the IL-2 only group, and significant expansion noted in the IL-2/IL-15 combination group. Expansion was also seen with the IL-2/EL08-1D2 combination; however, morphologically, these cells lacked cytoplasmic granules, a surprising finding, which was attributed to degranulation in culture [95]. Degranulation has been reported in cases of NK cell neoplasia, but NK cells are commonly associated with the presence of cytoplasmic granules. Finally, the authors reported 41% cytotoxicity at a 20:1 ET ratio for the cytokine-treated cell population. The EL-cultured cells showed lower cytotoxicity, and this was attributed to cell erosion in the co-culture system by 14 and 21 days [95]. Of note, the cytotoxicity that was reported in this study was lower than that of previous studies that did not perform CD5 depletion. This suggests that, in the absence of CD5 depletion, T cells and LAK/NKT cells are contributing to the cytotoxicity profile, likely by recognizing MHC-I on the surface of the CTAC cells [95]. Functional studies to this end were not pursued, as there was no anti-canine MHC-I antibody available at the time that could block canine MHC-NK interactions. 

Three studies have reported the use of recombinant canine (rc) interleukins, specifically rc-IL2 [69], rc-IL15 [97], and rc-IL21 ([59]) (Table 2). Foltz et al. described expansion of NKp46^+^ NK cells using either recombinant human (rh) IL-2 or recombinant canine (rc) IL-2. Lee et al. compared the use of rhIL-2 and rhIL-15 as compared to rcIL-15 alone. Shin et al. used rhIL-2 and rcIL-15 for their expansions and generated four treatment groups for rcIL-21: a control, a day 0–7 treatment, intermittent treatment (on the first day of each week), and continuous rcIL-21 treatment. 

In their study, Foltz et al. reported a significantly increased percentage and raw yield of CD3^−^NKp46^+^ canine NK cells when using rcIL-2 as compared to rhIL-2 [69]. The percentage of CD3^−^NKp46^+^ canine NK cells increased from 58% to 72.9% and the yield from 2.0 × 10^7^ to an impressive 9.58 × 10^7^ cells from 5 million canine PBMCs [69]. Lee et al. compared the rate of proliferation of non-B, non-T CLGLs between rhIL-2 alone, rhIL-2/rhIL-15, and rhIL-2/rcIL-15. The authors noted superior expansion in the rhIL-2/rcIL-15 group at 139-fold, when compared to the rhIL-2 group (32-fold) and rhIL-2/rhIL-15 group (91-fold) at the end of 21 days. Cytotoxicity against CTAC cells and IFN-γ production was also higher in the rhIL-2/rcIL-15 group [97]. Shin et al. also compared the proliferation of non-B, non-T CLGLs (CD5^−^CD4^−^TCRαβ^−^TCRγδ^−^) in each group. NK cells that were treated with rcIL-21 during the first week of the expansion only had superior expansion (90.6-fold) as compared to the intermittent (51.9 fold) and continuous (44.4-fold) treatment groups. The purity of the first-week group was 60.8–80.6% after 21 days in culture. In summary, each study demonstrated superior expansion with recombinant canine cytokines when compared to recombinant human controls [59]. 

## 5. Clinical Applications of Canine NK Cells

Clinical applications in dogs using NK cells have relied on ex vivo expansions, such as those described above. Two studies from Funk et al. examined the natural cytotoxicity of NK cells from healthy dogs and those with spontaneous neoplasms [98,99]. The authors isolated CLGLs from peripheral blood of controls and dogs with cancer by centrifuging lymphocytes onto microscope slides while using a cytospin centrifuge, applying a Giemsa stain and then identifying CLGLs morphologically. Following this, the isolated CLGLs were then stimulated in vitro with rhIL-2. Cytotoxicity was then measured against the CTAC cell line, with the controls performing significantly higher. This difference was attributed to a diminished proliferation capacity of NK cells from the cancer patients [98]. A second study investigated canine NK cells from 110 dogs with a variety of tumor types. In vitro cytotoxicity testing again demonstrated decreased natural cytotoxicity, particularly in dogs with mammary carcinomas [99]. Although these studies precede this review by 16 years, they were among the first to demonstrate aberrations in immune function in the setting of neoplasia in an immunocompetent translational model. We now know that the circulating NK cells may have a different phenotype than intra-tumoral NK cells, and this is important to keep in mind for future studies [5,7,47,82,100] Following this, the canine model was used to test the cytokine delivery therapies for safety and toxicity, such as liposome-DNA complexes encoding for the IL-2 gene to activate lymphocytes in vivo and inhaled liposome IL-2 to treat canines with pulmonary metastases [101,102]. Evidence of increased NK cytotoxicity following IL-2 administration was seen in each of these studies. 

With regards to cancer immunotherapy, Canter et al. performed a first-in-dog clinical trial utilizing canine NK cells, rhIL-2, and radiotherapy (RT) [44]. We expanded canine NK cells ex vivo while using various human cytokine combinations and genetically modified K562 feeder cells. We then demonstrated that the expanded NK cells had cytotoxic activity against a soft tissue canine PDX tumor in vitro as well as an in vivo NSG^TM^ mouse model. The expanded NK cells were noted to be effective against the tumor cells and an isolated cancer stem cell (CSC) sub-population [44]. Following pre-clinical mouse studies, we conducted a first-in-dog clinical trial in dogs with osteosarcoma (OSA). Dogs with locally advanced, non-metastatic OSA were given palliative RT and their blood was drawn for autologous NK expansion with rhIL-2. The dogs then received two intra-tumoral injections of NK cells. Dogs in this trial were found to have improved progression-free survival and decreased metastases when compared to the historical controls. The metastases control was thought to be due to the cytotoxic effect of NK cells on CSCs seen in the earlier canine PDX experiments, although we also observed increased granzyme B positivity in circulating CD45^+^ cells post-therapy suggesting the possible overall immunostimulatory effects of treatment [44]. Demonstration that the benefit of autologous NK transfer and RT persisted in the setting of the immunocompetent dog model is a prime example of the benefit of canine studies to bridge clinical translational from murine experiments to human treatment [44].

NK cells have also been studied in the context of the canine distemper virus (CDV) [62]. Following the expansion protocol described by Shin et al. above, [58] the CD4^−^CD21^−^ CLGLs exhibited dose-dependent cytotoxicity against both normal and CDV-infected cells. Notably, the cytotoxicity of against the CDV-infected cells was much higher than against the normal cells (54.7% vs. 42.5%). The expanded NK cells were noted to produce significantly high amounts of IFN-γ, and anti-IFN-γ neutralized the cytotoxic effects [62]. The CDV-infected cells were remarkable for the downregulation of MHC-I, likely removing inhibitory signaling that would normally restrain NK cell cytotoxicity. Finally, in dogs that were treated with anti-CDV serum, the antibody-dependent cellular cytotoxicity of the NK cells was enhanced. Therefore, the authors concluded that NK cells are important in controlling CDV infection and they have potential therapeutic applications in the management of CDV infection [62]. 

There has been considerable interest in extending the benefits of NK immunotherapy to cancer patients since the landmark treatment of metastatic cancer patients with autologous lymphokine-activated killer cells and rhIL-2 by Rosenberg et al. in 1985. Current practices for the treatment of human malignancies with NK cells include co-administration with chemotherapy to rescue natural cytotoxicity in the setting of tumor immunosuppression, or co-administration with immunoregulatory drugs, such as thalidomide, which activates NK cells via nuclear translocation of transcription factors [5,7,8,11,18,23,28,103,104]. Cytokine therapy, either given traditionally, conjugated to antibodies, or by engineering NK cells to express cytokines, remains a principal method for activating NK cells in vivo [28]. Finally, the concept of chimeric antigen receptor NK (CAR-NK) cells has been recently advocated as a novel application on the FDA-approved CAR-T therapy [10,11,105,106]. It is postulated that, when engineered with a CAR-receptor, NK cells will demonstrate more potent and durable cytotoxicity against tumor cells since NK cells function in the absence of MHC-I, which is a common immune evasion strategy of tumor cells. Additionally, as NK cells do not have the memory and lifespan of T cells, this might reduce some of the toxicities seen in CAR-T cell therapy [10]. 

However, several barriers have persisted in the last 30 years to the effective translation of NK cancer immunotherapy, and the majority of clinical trials have failed to progress beyond the early stage, demonstrating safety with limited evidence for robust efficacy [5]. As has been discussed, the source of NK cells, purification techniques, and expansion methods all influence the success of NK adoptive transfer. Low in vivo cytotoxicity and the survival of expanded NK cells has continued to derail successful clinical implementation [7,107]. Given the difficulties that are associated with initiating and conducting human clinical trials and given the key barriers to successful translation of NK immunotherapy to the clinic, companion dogs can serve as a valuable translational model to bridge the studies between mouse and man and further optimize the application of NK immunotherapy with the goal of realizing clinical efficacy. Better characterization is needed to more effectively understand the similarities and differences of dog NK cells with mouse and human. This will allow for the dog model to speed clinical translation of NK immunotherapy and overcome key barriers in the optimization of NK cancer immunotherapy, including trafficking, longevity, and maximal in vivo support. 

## Figures and Tables

**Table 1:** Phenotypic Surface Markers of Canine NK Cells based on Current Evidence.

**Known Canine NK Cell Activating Receptors**				
**Receptor**	**Gene**	**Verified by**	**Additional info**	**Homology**
CD5^dim^	CD5	Flow cytometry	15% of PBMCs	Human, mouse
NKp46	NCR1	Flow cytometry	2.5% of PBMCs	Human, mouse
CD16	FCGR3A	DNA Sequencing	Absent/not annotated on CanFam3.1 assembly	Human, mouse
NKG2D	KLRK1	DNA Sequencing	Annotated on CanFam3.1 assembly	Human, mouse
CD3	CD3E	Flow cytometry	Typically a T-cell marker, persists in candidate populations of canine NK cells	Human, mouse
**Known Canine NK Cell MHC-I Inhibitory Receptors**				
**Receptor**	**Gene**	**Verified by**	**Additional info**	**Homology**
Ly49	Ly49	DNA sequencing, Southern blot	Cysteine-to-tyrosine mutation present, function unknown	Mouse
CD94	KLRD1	Flow cytometry	7% of PBMCs. Function unknown, lack of NKG2A to form heterodimer	Human, mouse
KIR	Absent	DNA sequencing	LRC appears to be truncated prior to KIR gene locations	Human

**Table 2:** Recombinant Canine Cytokines for ex vivo NK Expansion.

Study	Year	Starting Population	Feeder Cells	Media	Additives	Human Cytokines	Canine Cytokine		Days
Foltz	2016	CD3^−^/ NKp46^+^ cells from healthy dog PBMCs	Irradiated K562 Clone9. mbIL-21 cells			100 IU/mL rh-IL2 *	6.1 ng/ mL rcIL-2 *	Not used in combination with human cytokines	21 days
Lee	2015	Healthy dog isolated PBMCs	100-Gy-irradiated K562 cells	RPMI	fetal bovine serum, penicillin, streptomycin	100 IU/mL rhIL-2 10 IU/ml rhIL-15	10 IU/mL rcIL-15	Used in combination with rhIL-2	21 days
Shin	2015	Healthy dog isolated PBMCs	100-Gy-irradiated K562 cells	RPMI 1640	fetal bovine serum, penicillin, streptomycin	100 IU/mL rhIL-2 10 IU/mL rhIL-15	5 ng/mL rcIL-21	Used in combination with rhIL-2 and rhIL-15	21 days

* rh = recombinant human, rc = recombinant canine; CD, PBMCs, RPMI,
